# “The Opposite of Treatment”: A qualitative study of how patients diagnosed with psychosis experience music therapy

**DOI:** 10.1080/08098131.2014.890639

**Published:** 2014-03-03

**Authors:** Hans Petter Solli, Randi Rolvsjord

**Affiliations:** ^a^ Lovisenberg Diakonale Hospital, Oslo, Norway; ^b^ GAMUT, The Grieg Academy, University of Bergen, Bergen, Norway

**Keywords:** music therapy, mental health, psychosis, recovery, user-perspective, agency

## Abstract

Previous research studies regarding music therapy and severe mental illness have mainly adopted quantitative methodologies in order to study the effectiveness of music therapy interventions. Studies that have explored service users’ experiences of participation in music therapy are small in number, and almost nonexistent in the field of psychosis. This study aimed to explore how mental health patients with a diagnosis of psychosis experienced participation in music therapy, in general, and more specifically how they experienced music therapy in relation to their current mental state and life situation. Nine inpatients with psychosis were interviewed using a semi-structured interview focusing on the participants’ experiences of music therapy in individual sessions, groups, and performances. Through the use of interpretative phenomenological analysis, four super-ordinate themes central to the participants’ experiences were found: freedom, contact, well-being, and symptom reduction. Based on the findings, mental health recovery, positive mental health, and agency are proposed as constituting a better framework for music therapy in mental healthcare than a primary focus on symptom remission and functional improvement.

## Introduction

Psychosis is characterized by crucial changes in thoughts and perceptions as well as in a person’s emotional and social life. The lifetime prevalence of all psychotic disorders has been found to be up to 3%, and around 1% for schizophrenia (Perälä et al., 2007). Despite promising developments in medical treatment and psychosocial rehabilitation, there are growing calls for other, more effective approaches to help and support people with psychosis (Bentall, 2009; Fledderus, Bohlmeijer, Smit, & Westerhof, 2010; Herrman, Saxena, & Moodie, 2005; Slade, 2009). Within the last decade, outcome studies have documented positive effects of music therapy on symptoms and functioning for people diagnosed with schizophrenia and the wider condition of psychosis (Gold, Solli, Krüger, & Lie, 2009; Gold et al., 2013; Mössler, Chen, Heldal, & Gold, 2011). As a consequence of this emerging evidence base, music therapy is now being recommended in national treatment guidelines for people with psychosis in UK and Norway (National Collaborating Centre for Mental Health, 2010; The Norwegian Directorate of Health, 2013).

However, while an increasing number of such quantitative outcome studies have documented the effectiveness of music therapy in regard to various standardized outcome measures (mainly measures of symptom reduction), the service user-perspective of these patients^1^ ^1^In this text the term *patient* is used because the music therapy takes place in a hospital setting. Additionally, the terms *user* and *participant* are applied when referring to research and general mental health-care contexts. has been explored to a much lesser extent. Recent qualitative studies involving people with severe mental illness have primarily focused on development of theoretical and clinical frameworks (De Backer, 2004; Odell-Miller, 2007; Pedersen, 2006), and case studies and case reports have mainly addressed the therapy process from the music therapist’s perspective (e.g. Bruscia, 2012; Jensen, 1999; Metzner, 2003; Næss & Ruud, 2007; Odell-Miller, 1991; Silverman, 2003; Solli, 2008). Some surveys and questionnaires have been conducted (e.g. Baines, 2003; Baines & Danko, 2010; Choi, 1997; Eyre, 2011; Reker, 1991; Silverman, 2006), but their predetermined focus has resulted in limited opportunities for exploring service users’ own perspectives. A few personal narratives written by users can also be found (Hibben, 1999; Tyson, 1981).

Existing research studies containing first-hand accounts of users’ experiences in music therapy are either conducted with informants who have nonpsychotic illnesses (Carr et al., 2011; Rolvsjord, 2010; Stige, 1999, 2012a) or with a broader group of mental health clients (Ansdell & Meehan, 2010; Baines & Danko, 2010; Clemencic-Jones, 1998; Grocke, Bloch, & Castle, 2009; Hammel-Gormley, 1995; Jampel, 2007). First-hand accounts from patients diagnosed with psychosis can be found in Moe’s (2000; Moe, Roesen, & Raben, 2000) doctoral thesis, but the study is limited to the guided imagery and music (GIM) method. Finally, Silverman’s (2010) mixed-method study contains a small section where participants with psychosis have commented upon sessions in interviews. To our knowledge, a qualitative in-depth study on how patients with psychosis experience music therapy in general has not previously been conducted.

The limited interest in the user perspective found in music therapy may reflect a general trend in research literature on mental illness, where mental health service users’ subjective experiences have been neglected (Jenkins & Barrett, 2004; Strauss, 2008). Critical voices claim that this lack of first-person accounts has resulted in the silencing of a whole group of people who have thus been left subject to the perspectives of others (Geekie, Randal, Read, & Lampshire, 2012). While nomothetic, generalized knowledge has been prized, this provides us with only “half the story” (Slade, 2012). Consequently, there is a need for more research which can contribute idiographic, subjective knowledge. Psychosis is, first and foremost, a human experience, in the sense that the psychotic experiences are only immediately accessible to the person who is psychotic (Geekie et al., 2012; Slade, 2012). Research on users’ experiences is therefore able to contribute to the overall scientific quality of research related to severe mental illness (Strauss, 2008).

During the last decade, attention has increasingly been paid to user perspectives from people experiencing mental health difficulties, with these perspectives being accorded value across health care politics, research, and practice (Thornicroft & Tansella, 2005). This development is closely related to the growing influence of the notion of *mental health recovery* ^2^ ^2^Related terms frequently used in literature include *personal recovery* (Slade, 2009), *recovery in mental illness* (vs. recovery from mental illness) (Davidson et al., 2009), and *social recovery* (Repper & Perkins, 2003; Tew et al., 2012). (Anthony, 1993; Slade, 2009). Mental health recovery is a critical and user-oriented paradigm that has arisen from first-person accounts from people with mental health difficulties, contributing rich “insider” perspectives on what it is like to live with a severe mental illness, and what helps and hinders processes of recovery (Davidson et al., 2005; Deegan, 1996; Read & Reynolds, 1996). This knowledge has led to radical changes in the understanding of mental health and illness and in the content of mental health services being proposed, and is increasingly permeating international mental health policy (Slade, Adams, & O'Hagan, 2012). A much cited definition describes recovery as:

a deeply personal, unique process of changing one’s attitudes, values, feelings, goals, skills and/or roles. It is a way of living a satisfying, hopeful, and contributing life, even with limitations caused by the illness. Recovery involves the development of new meaning and purpose in life as one grows beyond the catastrophic effects of mental illness. (Anthony, 1993, p. 7)

This notion of mental health recovery differs radically from *clinical recovery*, especially regarding the significance of symptoms. While clinical recovery refers to full symptom remission (Liberman, Kopelowicz, Ventura, & Gutkind, 2002), mental health recovery is less about the cure and treatment of illness, and more about the challenges and possibilities of living with various degrees of illness and problems. Additionally, the process of recovery is linked to a higher degree with contextual and social aspects of a person’s life, where social relationships, social roles, and social inclusion are seen as crucial elements for a better life (Repper & Perkins, 2003; Tew et al., 2012; Topor, Borg, Di Girolamo, & Davidson, 2011). Essentially, mental health recovery highlights how people with mental health difficulties must be considered responsible and active agents in their own life and recovery process. Thus, the professional’s engagement related to users’ recovery is closely linked to the promotion of self-determination, hope, and community inclusion (Davidson, Tondora, Lawless, O’Connell, & Rowe, 2009).

The recovery orientation has clear similarities to community music therapy and resource-oriented perspectives in music therapy. However, the notion of recovery has not gained much attention in music therapy literature, although within the last few years there seems to be an growing interest (Chhina, 2004; Grocke, Bloch, & Castle, 2008; Jensen, 2008; Kaser, 2011; Kooij, 2009; Maguire & Merrick, 2013; McCaffrey, Edwards, & Fannon, 2011; Solli, 2009, 2012; Solli & Rolvsjord, 2008). In a meta-synthesis of studies of service users’ experiences of music therapy (Solli, Rolvsjord, & Borg, 2013), a central finding was that music therapy contributed to many of the central aspects found important for a person’s recovery process. However, the data material on which this meta-synthesis was based varied in quality and richness, and contained few participants with psychosis.

This article reports on a study which features data provided by mental health patients with ongoing psychosis and analyzed using interpretative phenomenological analysis (IPA). The primary purpose of the study was to gain first-hand accounts from people diagnosed with psychosis who were participating in music therapy. This article addresses: (1) what it is like for inpatients with psychosis to participate in music therapy; and (2) how participants with psychosis experience music therapy in relation to their mental health and current life challenges.

## Method

### Participants and setting

Prior to data collection, the study received ethical approval from the Regional Committee for Medical and Health Research Ethics, and was reported to the local health and social services ombudsman. Nine participants were recruited from the first author’s music therapy practice at a closed inpatient intensive psychiatric unit at a hospital in Norway. Selection of participants was done purposively, choosing people who were expected to offer insight into the research question (Smith, Flowers, & Larkin, 2009). Participants had to meet the following inclusion criteria: (1) diagnosed with a psychotic illness, and/or have experienced psychosis within the past year; (2) motivated for music therapy; and (3) verbally capable of expressing her/himself in an interview. Patients in an acute psychotic phase were excluded or included once acute symptoms had been ameliorated. Criteria for inclusion and exclusion were assessed by the researcher and a psychiatrist or psychologist, based on information collected by the clinical team on the ward. A letter of informed consent was signed by all participants. Three interviews with each patient were intended, but due to practical factors such as sudden discharge and rapid changes in mental condition, an unequal number of interviews were completed with each patient. All participants in the study had a strong interest in music, although this was not a criterion of inclusion. For more characteristics of the participants, see Table 1.

**Table 1.** Participant information.

Gender	Female	4
	Male	5
Age in years	Range	21–41
	Mean	32
Diagnosis (ICD-10)	Paranoid schizophrenia	7
	Schizoaffective disorder	1
	PTSD and undifferentiated psychosis	1
Hospitalization§	Compulsory	8
	Voluntary	1
Contact with mental health services in years	Range	2–20
	Mean	10
Duration of music therapy in months	Range	3–34
	Mean	13
Number of sessions	Range	14–55
	Mean	31
Music therapy format	Individual sessions only	P2, P6
	Individual sessions and groups	P4, P7, P8
	Individual sessions, groups, and performances	P1, P3, P5, P9

The music therapy offered to participants in this study can be characterized as humanistic (Ruud, 2010), resource-oriented (Rolvsjord, 2010), and with influences from community music therapy (Stige & Aarø, 2012). Each participant was offered a 30–60-min weekly individual session in a music therapy room, a weekly 45-min open group session in the ward TV-room, and opportunities to perform music at the hospital’s “season parties” (see Table 1 for actual participation). The therapeutic collaboration involved a wide variety of musical engagement, based on the preferences of the participants, but including structured and free improvisations, playing and singing from song-books, learning to play instruments, music listening, song-writing, recording and mixing of music, production of CDs, and uploading songs to the Internet. In addition, verbal conversations were a natural part of all sessions.

## Data collection

A semi-structured interview guide with open questions was developed through discussion between the present authors (Kvale & Brinkmann, 2009). The schedule was intended to capture a wide range of the participants’ subjective experiences within music therapy, both musical and relational. The two initial questions were: “What kind of role does music play in your life?” and “Can you tell me about your experiences with music therapy the last couple of weeks?” A flexible and nondirective interview approach was adopted in order to enable the participants to express their experiences in ways that best suited their personal narrative style. Strategies for minimizing the level of stress for people with cognitive and verbal challenges whilst maximizing the quality of the interviews was used (Kirkevold & Bergland, 2007). Participants were interviewed individually by the first author in durations of 15–40 min adjusted to their mental and emotional state and response. As a rule, the interviews were done in separate sessions, but due to the participant’s mental and emotional states some interviews were conducted in the continuations of the music therapy sessions. All interviews were recorded and transcribed.

The double role as therapist and researcher was experienced as advantageous in order to lower the stress level of the participants, and in gaining a deeper understanding of their context. However, this double position called for a reflexivity (Finlay & Gough, 2003; Stige, Malterud, & Midtgarden, 2009) and review of attitudes and positions throughout the project. The use of reflexivity notes, clinical supervision, and regular conversations with the research supervisor (second author) was important in this process. Alternation between the closeness that gave access to vital knowledge and the reflection that allowed critical thinking was of great importance. It was also important to be reflexive about possible tendencies for participants to withhold negative experiences and exaggerate positive aspects when being interviewed by their therapist.

## Analysis

The data was analyzed using IPA, as outlined by Smith et al. (2009). The goal of the analysis was to learn something from the participants’ psychological world, and to try to understand the meaning of the content by engaging in an interpretative relationship with the text. First, each interview transcript was read a number of times and relevant parts were marked. Remarks and initial codes were noted in the right-hand margin, using the software Atlas.ti. After several rounds of reading transcripts while editing and developing emerging codes, there was a process of grouping codes into associated families or clusters. Then, code families that were closely connected were merged and renamed, before the clusters most prominent in terms of illuminating the research question were chosen as key themes. The final step of the data analysis was the writing phase, where the selection of extracts for each theme was processed into larger parts of text. At all stages of this analytic interpretative process, there was a constant alternation in focus, moving back and forth between details in the text, the wholeness of each interview, the sum of all interviews and the project as a whole, as well as various theoretical perspectives, often referred to as the hermeneutic circle or spiral (Alvesson & Sköldberg, 2000).

## Findings

Four super-ordinate themes were drawn from the analysis, as presented in Table 2.

**Table 2.** Key themes and sub-themes.

Theme 1: **Freedom**	Freedom from illness
	Freedom from stigma
	Freedom from treatment
Theme 2: **Contact**	Contact with oneself
	Contact with aliveness
	Contact with emotions
	Contact with other people
Theme 3: **Well-being**	Enjoyment and satisfaction
	Motivation
	Mastery
	Hope
Theme 4: **Symptom relief**	The psychotic state
	Disturbing thoughts and voices
	Visual hallucinations

## Theme 1: Freedom

*Freedom from illness:* Most of the participants stated some kind of ambivalence in relation to music and music therapy being connected to their diagnosis and treatment of their condition. Rather, they described music therapy as a place where they managed to take their mind off the illness:

P1: There’s a freedom from all possible illness, then… and psychosis and everything that’s bothersome. There’s something good and creative in this room.

HP: Did you notice that when you came into the room now?

P1: Yeah, I mean I've always noticed that it's good to be here. It’s peaceful, you know. It’s peaceful in this room. There’s no disease in this room. There are no negative spirits here, somehow. There’s peace.

The everyday situation at the ward was described as extremely demanding, both because living in a closed ward together with other people in crisis was a challenging experience, but also because major parts of the treatment focused strongly on their diagnosis and deficits. In this context music therapy was experienced as a sanctuary, a setting that afforded experiences of freedom and peace, or at least some distance from their everyday struggle.

I haven’t had the feeling of mastering so much while I’ve been here at the hospital. It’s mostly been complicated stuff that I’m supposed to relate to … and learn from, so I don’t end up where I started, sort of thing… so I don’t end up at the doctor's office again. But I’ve sort of forgotten about that when I play. I don’t think about those things. I suppose I think less and less about the difficult things the more I play…or the more I listen to music… (P9)

Several participants expressed skepticism about the idea of music being used as treatment, as if this would be a way of intruding into one of the few illness-free zones they had left.

I think that it [music therapy] should be an area that is free from analyzing and stuff. It should just be about music. Not necessarily what … kind of diagnosis you have. You shouldn’t have to talk about such things here. I think that’s the best, you know… (P4)

In contrast, one participant (P3) expressed that she would like to discuss her psychotic experiences more frequently in music therapy.

*Freedom from stigma:* The staff’s general focus on illness at the hospital was often experienced as overwhelming and intense, leaving some participants with a struggle of identity, fighting against the stigmatic role of being a “psychiatric patient”. In contrast, music therapy was experienced as helpful in rebalancing self-perception, enabling participants to discover or reawaken their own interests and abilities. Being able to demonstrate their musical resources to other patients and staff, whether via music therapy groups, performances, or recorded CDs, was experienced as a means of fighting stigma.

Once I come here [to the hospital], I’ve got “sick” written on my forehead. And then there’s lots of staff-members who talk to me like I'm really sick and stuff. (…) Because I often feel like a misfit, that there’s sort of so much wrong with me, you know? And when I'm here [in music therapy], then I don’t feel that there’s so much wrong. (P3)

*Freedom from treatment:* Most of the participants said that they enjoyed the fact that music therapy sessions did not focus strongly on their illness and problems: “Because there’s enough of that elsewhere” one woman remarked (P2). Paradoxically, music therapy was often experienced as a break from treatment:

HP: Did you think that music therapy was a part of the treatment you got here, or did you think of it as something else?

P7: Hmm … I suppose I thought of it as something different … actually. In fact, I think of it almost the opposite way. It’s not a part of the treatment, it is an alternative to treatment. It is something else … more like a break from treatment.

Most of the music therapy processes did include engagement with difficult themes and emotions in the patient’s present situation. However, music therapy was experienced as handling these themes differently than other treatment approaches at the hospital. A woman whose individual sessions included the writing and recording of a song about her self-harming reflected:

I think I got to express some thoughts and stuff without necessarily having someone trying to fix it. Just see how it is…(…) Often when someone talks … or if I say something … almost always someone will suggest what I should do with it, or give me extra pills, or whatever… Whereas when we made up [a] song, then it wasn’t … there wasn’t any answer… it was just a sort of expression. (P2)

## Theme 2: Contact

*Contact with oneself:* Music was described as a personal matter by all of the participants. Accounts suggested that some music was experienced as touching closely on personal and social identity, and that music could provide a strong sense of being oneself and having an inner core.

For me it [the music] … is therapeutic because it … it hits a nail within myself… in a way, that tells me… that the music hits me in a way … that gives me a feeling of that…I belong in the music, this is me, the music is me – exactly the music I hear now, it’s me. (…) I find a real sense of identity in music. And a feeling of being home… and personal … personal … re-involvement in myself … every time I listen to music. (P6)

Music therapy was also connected to positive change and development regarding participants’ own identity, self-awareness, self-confidence, and self-esteem.

I feel I've become a different person after having these sessions. Because it's … I feel like I've been more like… Before I was a bit like shy and stuff… and I was maybe not that present and stuff. But after these sessions, I felt I’d really got more guts inside me. (P3)

*Contact with aliveness:* Experiences related to a sense of aliveness and presence were reported by several of the participants. These sensations were described as being manifested through bodily sensations, “sparkling” feelings, experiences of taking up space in the room, and awareness of becoming “a new human being”.

HP: How does it feel to sing it, then?

P8: It's like being in love. It tickles in my stomach and it …. My heart beats and I feel the pulse in my fingers and in my body and I feel alive, happy for life, and full of emotions and strength, and that I’m mastering something, and that I’m expressing something … that I really mean.

One of the participants experienced loud improvisations on a drum kit as enabling her to become more alive and aware of her body and breath, helping her to separate herself from the rest of the world – experiencing sensations similar to when she cut herself with razor blades.

I don’t know my body that well … it's sort of numb. So the only thing I can do, then, is to cut myself, because then I can feel my body. (…) But as soon as I start to beat the drums then …there is something that works (…) I feel … you know … a little bit … actually a bit the way I feel when I have harmed myself. That sort of … well … I'm here, sort of [touches her hand]. Here it ends in a way, and here it begins. (P2)

*Contact with emotions:* Music as a promoter of emotional contact was highlighted by all participants and constituted the greatest number of coded sections. Accounts pointed toward music therapy as an arena where emotions (and feelings) are noticed, created, developed, and expressed.

Music is releasing. It can bring so many emotions. (…). Creates feelings. Gives feelings. It is feelings. So that you get feelings. You get new feelings, get inspired. Or just feel something or think something, reflect a bit, think something new, change yourself, get associations, immerse yourself in something, remember … have memories … (P8)

Music was highlighted as a particularly good way of connecting with, and expressing, emotions connected to pain, anger, and aggression. Using swearing and rude words in song-lyrics and raps, and hitting drums, and playing on distorted guitars, were given as examples of ways of getting in contact with and expressing these emotions. Performing and producing CDs provided opportunities for communicating emotions and experiences to other people both inside and outside the hospital.

Because I… I feel… when I sing and stuff, then I get a lot out… like anger and dirt, in a way. So I feel I’ve become more like conscious, then, and … stronger … (…) I feel it has helped me very much to dare to say “Fuck you, Child welfare!” and show it to others. You know, to show them that I'm aggressive and angry because someone’s done something wrong. And that… that what they’ve done is not right, in a way. (P3)

*Contact with other people:* That music was a way of making contact with other people was also a major finding in this study. Musical interplay was experienced as an arena where contact was important in order to make the music flow and groove. Musical and social relationship with the music therapist was experienced as an important first social step, and for some of the participants an eye-opener that made them more aware of the importance of being social.

When I'm alone then it [the rap] doesn’t work at all. But when I'm here, then I always come up with something. (…) When you're sitting there with the drums, then something … it's like something … I’m rapping. If you left, the rap would disappear, really. But when you come here, then it's there, right? (P1)

I try to look at you sometimes then to see where I am… and see what you think… about where we are. (P6)

Several participants talked about music as a way of connecting to the world outside the hospital. Some of them had experienced giving a CD with their own recordings to other patients, staff, friends, or family as a way of connecting socially or maintaining social relationships with others. Accounts also included making friends at public concerts, joining a choir or band, uploading music to the web, and chatting with people who listened to it, or trying to get a gig.

I was out a couple of days ago. Then I went into a pub. So I started talking to a guy there, you know. Then it turned out he was the man responsible for the music, you know. So he got my email and my phone number. (…) Maybe I'll get a gig, then. (P5)

## Theme 3: Well-being

*Enjoyment and satisfaction* were the most prominent aspects referred to by participants in this study. Many of the participants used words like *fun*, *amusing,* and *happy* to describe their participation in music therapy.

No, I think it's been fun, pure and simple. (P4)

It’s been a rush of happiness really. It’s been positivity all the time. There’s not been anything negative, you know? That struck me as a bit unusual … (P9)

Being admitted to a closed ward for several months was referred to as inactive, boring, and de-motivating. In this context the enjoyable qualities of music therapy were highly valued.

So it [music therapy] has been positive in that phase where I was stuck in the hospital, then. I didn’t have much else to do here. At that time I had nothing else to do at the hospital. Just walked back and forth in the hallway, and ate and slept and didn’t do much else. It filled the day a little bit anyhow. Made it a better stay. (P9)

Yes, the music is something that pulls me up. Music pulls me up. It's therapeutic for me. It’s a pity I don’t do it more often because I benefited from it. I get very much pleasure from it. (P6)

Although participants highlighted that music therapy made them feel better, more satisfied, and healthy, music therapy was not experienced as diminishing all worries and problems, but rather as bringing more wellness into life despite the challenges caused by the illness.

I’ve felt very well when I've been here [in music therapy], you know. Even though I have seen things and stuff. But I've felt very healthy and well, you know. (P3)

*Motivation:* A majority of the participants talked about music therapy as a motivating activity, something they looked forward to and therefore managed to attend, even when having a bad day. Often music therapy was highlighted as being more motivational than other forms of therapy at the hospital, and a general complaint was that one session a week was too little.

I think it’s much easier to wake up to music therapy than to other boring stuff. Conversations and stuff, I’m almost not capable of that. (P3)

I've been looking forward to it. It's the only thing I've been looking forward to here. (P8)

As a majority of the participants were compulsorily detained at the hospital, their motivation for receiving help was initially quite small. However, music therapy was described as a service that contributed to reconsideration of their resistance against hospitalization. One woman said that if she could have had music therapy every day, she would have admitted herself voluntarily (P8). Another participant said:

Yeah, I really mean it. If it weren’t for the music therapy, I wouldn’t have been able to stand being here. Then I would have run off long ago, maybe… (P5)

Some of the participants compared music therapy to other forms of therapy, conveying that they found music therapy more motivating because of its physical, active, social, and enjoyable nature.

You get the chance to develop a part of yourself, not just be passive. Because I often find that a lot of the verbal treatment, conversations with the doctor and psychologist, it’s very passive, you …become very superficial and very passive. (P7)

*Mastery:* Most participants talked about experiences of mastery in music therapy. These experiences were often related to their own sensations of coping with an instrument or a piece of music, but were also connected to reactions from other people during a group session or a performance.

I think I mastered the tam-tam drums. Like Mona [a fellow patient], she came up to me and said “you're really good at playing”, you know. That’s nice. So I try to be nice in return. It’s positivity. It leads to something positive. (P9)

Recordings and performances experienced as successful by the participants were reported to give sensations of mastery, to promote feelings of wellness and afford better self-esteem:

To succeed, or be able to complete a project, you know … that makes you feel better automatically. That it is *you* who has done it, and that it was actually good, and that others like it … then you’ve done something that points in the right direction. (P5)

In contrast, two of the participants in this study had negative experiences of their not having achieved mastery when listening to recordings of themselves. For one man this was very hard to cope with:

HP: When you played the saxophone – it was not just positive for you?

P6: No, I was admittedly very poorly prepared then. But I had no joy, there was nothing … I thought it sounded insanely better when I did it myself: then when you recorded it, I got a fucking disappointment, that is. (…) I know for myself that I sounded better than that … I know that because I used to take lessons. (…) It was a *heavy* disappointment. I became very depressed.

*Hope:* It was evident in this study that participation in music therapy was connected to experiences of hopes and dreams for many participants. In a life situation described as full of obstacles, mental health threats and pain, music and music therapy were connected to an amplified hope for a better future.

So I feel that it gives me huge joy and lots of hope. I somehow look forward [to the future] when I'm doing music. (P3)

Several of the participants spoke about their plans for continued musical engagement, for playing and performing music after discharge. Some plans were perceived as more realistic, while other statements were interpreted as dreams or wishes with more unrealistic outlooks. However, they all depicted music as a strong facilitator of hopefulness.

Yes, it’s my dream to work as an artist and make money out of it. And make a living of it. Maybe do some concerts now and then, and… some gigs, who knows? (P5)

Having a hobby that theoretically has the potential of bringing success and fame, no matter how unrealistic that dream may seem at the moment, was an aspect of playing music that was inspiring for one participant:

I'm thinking, damn, I may not be the next Madonna, but I'm not going to be the next Madonna … I do have an alter ego name, though… But I… You do want to have a hobby, and then you sometimes get some stars in your eyes. But it's fun to dream! And maybe you can develop it one day into something big (…) But now I’m on social security, so I can keep on with it on my spare time. (P8)

For one participant, music was experienced as so important that he could not imagine managing life without it, referring to music as being the essence, nourishment, sound, hobby, and creativity in his life.

It [the music] has saved me many times, I guess…that I've always had a hobby. (…) Creating something instead of being destructive, kind of. (P5)

## Theme 4: Symptom relief^3^ ^3^To some degree, themes 2 and 3 could also be related to the relief of symptoms, particularly negative symptoms. This category is to a larger degree about psychotic experiences, also referred to as positive symptoms


*The psychotic state*: Many participants did not want to go into detail about their psychotic experiences and how these related to music. Several reasons were given for this: that, as psychotic experiences were not a common theme in music therapy sessions, it felt strange to start talking about them in the interviews (P7); that psychotic experiences were so complex that a lot more time would be needed to explain them (P6), or that psychosis or illness should not be a part of music therapy at all (P4). These responses can clearly be linked to the issue of freedom, as described under theme 1. For participants who shared their accounts about how music and music therapy related to the condition of psychosis, each story had a personal and unique character. One participant said that, in general, treatment had made her become sceptical toward the reality of the world around her, but that music therapy was different.

P2: What's scary with this ….. to be told that you have this kind of diagnosis, is that suddenly you can’t rely on the world, you can’t rely on yourself, because it’s not all that is there for real (…)

HP: Does music therapy do anything in relation to … to it, sort of, you being more sceptical or less sceptical, in a way?

P2: No, what’s nice, maybe especially, is that here it doesn’t matter if it’s true or not, it … it's good anyway. It doesn’t matter to me if it’s real or not.

One participant said that music had a positive and transformative impact on what he called “the spell”:

HP: How is it to play music or listen to music?

P6: It can amplify the spell in a positive way. The psychosis may become pleasurable and with, with, with … it may seem … like … a healthy nerve activity (…) compared to a negative nerve activity as psychosis is often found to be, you know, for humans…

In contrast, one of the participants had no experience of music affecting psychotic experiences. When asked if music therapy and music therapy were helpful for her in relation to her illness and health, she connected music to emotional states, but doubted that it could influence psychosis in any way (P7).

*Disturbing thoughts and voices:* Participation in music therapy was described as having a vital impact on what were referred to as thoughts by some and as voices by others. These thoughts or voices were often characterized as threatening, destructing, and disturbing, and were constantly present in the participant’s everyday life. In particular, active music playing and improvising on instruments were reported to moderate or even eliminate annoying thoughts and voices whilst playing, and sometimes for a few hours after a session. Playing music together with the therapist was reported to be better than listening to music at home.

I've stopped thinking destructive thoughts, at least when I've been playing music, then. (P9)

Patients’ explanations of why music and music therapy were helpful included calming down, being distracted, concentrating, and refocusing.

It’s one of the few things that really helps me get rid of these driving thoughts. Maybe it’s just about focusing on something else. Calming down and focusing on something else, simply. You follow the music instead of grinding round and round in your head all the time. (P2)

One participant said that playing loud music helped her to drown out what she experienced as loud and annoying sounds around her.

P2: The beauty of playing drums is that it drowns out, in a way … all the annoying sounds that bother me otherwise, as…

HP: Yes. Voices that you hear, or..?

P2: Yes, sort of … kind of, the way that clock [points at the clock on the wall], it becomes difficult to concentrate … when it keeps on like that…

HP: Because you hear it very loudly?

P2: I hear it very well. Also the hiss from …

HP: From the radiator?

P2: Yes

HP: So the drum set really takes up all the space?

P2: Yes, it feels so good!

Only two participants talked unreservedly about hearing voices (P1 and P3). One participant reported that music was so important to her that she gained strength to battle and take control over the voices.

HP: What happens with the voices then, when you're doing music?

P3: With my voice?

HP: No, the voices you hear … you just said that you hear voices inside your head. What happens when we’re singing and stuff down here?

P3: No, then I say like: Fuck you! Now it's my turn!

HP: Really?

P3: Yes!

HP: Do they say something back, then?

P3: Yes, they say: “No. You listen!” And I just say: “No, fuck you, it's my turn now!”

HP: And then you sing?

P3: Yes!

*Visual hallucinations:* One participant talked about seeing things in the room that she knew were not real, usually referred to as visual hallucinations (P3). For this woman, improvising on the drum kit together with the music therapist on bass guitar became a way of battling what she first experienced as shadows and later called “dead people” present in the room. In an interview done immediately after a session she explained how such an improvisation on drums and bass had had an impact on her visions.

HP: How was it just now when we came into the room and talked together?

P3: Well, I had a belief that I have become a spirit. So I had to help those in the church cemetery. And just then I saw a dead body … who was sitting behind you. But when I started playing and so on then I started thinking about other things, and then suddenly I looked there, and it was gone.

HP: What was it that happened then, do you think?

P3: I don’t know, but I think my brain wanted to focus on something else because I was thinking very strongly that I had to get rid of this image.

## Discussion

Our analysis highlighted four super-ordinate themes central to participants’ experiences in music therapy: freedom, contact, well-being, and symptom relief. The overall impression of our findings is that music therapy is highly appreciated amongst patients, and is experienced as positive and supportive in relation to their life situation and health in multiple ways. In this respect, we may say that the users’ perspective outlined in this article does not diverge from previous findings in outcome studies of music therapy for patients with psychosis, but rather confirms previous accounts of the effectiveness of music therapy. However, as will be discussed, our findings, and in particular those connected to the “freedom” theme, at the same time challenge and even question the role of music therapy as part of treatment provision.

A prominent finding was that music therapy was experienced as engaging, motivating, and enjoyable by all of the participants. Psychotic illness (i.e. schizophrenia) is in general characterized by apathy, lack of motivation, and emotional flatness (Faerden et al., 2009), referred to as negative symptoms, or as a necessary coping strategy (Boevink, 2012). These are serious aspects of psychotic illness which prognosticate poor functioning and quality of life (Katschnig, 2000), and where standard treatments show limited progress (Kirkpatrick, Fenton, Carpenter, & Marder, 2006). Given these premises, the participants’ reports of how music therapy made them feel more vital, uplifted, joyful, hopeful, and motivated, and enabled them to become more active participants in their everyday lives, constitute an important and promising finding. Here too, we may say that the present study confirms results from outcome studies with regard to the significant effects of music therapy on negative symptoms for people with severe mental illnesses (Gold et al., 2009, 2013; Mössler et al., 2011), although in our findings such aspects were linked to experiences of well-being (that is, to an increase of positive health) rather than to a reduction of negative symptoms. In the light of the users’ experiences, we may therefore suggest that it is more appropriate to describe recovery processes in terms of the level of positive health (i.e. as described by Provencher and Keyes (2013) on a continuum from languishing to flourishing) than in terms of symptom reduction.

A second prominent finding was the participants’ experiences of being in contact. The fact that some of the participants joined group music therapy while other did not (see Table 1) could be a factor that affected how music therapy’s role in facilitating such contact was perceived, but no clear tendencies were identified. Experiences of contact may be discussed in relation to a vital but undervalued aspect of the development and character of psychosis: the subjective experience of alterations of the first-person dimension (Davidson & Strauss, 1992; Lysaker, Buck, & Lysaker, 2012). Such alterations are described as “a sense that I had lost myself, a constant feeling that my self no longer belonged to me” (Kean, 2009, p. 1034). This lost or weakened capacity for intrapersonal and interpersonal dialogue, often referred to as ipseity disturbance (Pérez-Álvarez, García-Montes, Vallina-Fernández, Perona-Garcelán, & Cuevas-Yust, 2011), was also reported among participants in our study. Here, music was experienced as something that was closely connected to identity and a sense of having an inner core, whilst music therapy was reported to help in regaining contact with a sense of self, identity, and aliveness. With a missing vital presence in one’s own life, interpersonal contact becomes overwhelming: hence a diminished personal identity and sense of self are closely connected to social agency (Lysaker et al., 2012). We found that many participants related music therapy to a renewed motivation for being in contact with others. These findings suggest that music therapy is an approach that can meet some of the core challenges for people who are experiencing psychosis in a promising way.

In general, there is a lack of knowledge about potential harmful consequences in art-based therapy for patients with psychosis (Ruddy & Milnes, 2005). In our study seven patients reported no harmful or negative experiences related to their participation in music therapy. The two remaining participants, who in general found music therapy helpful, reported negative experiences from listening back to audio recordings of their own musical performance. They both expected their singing or playing to sound qualitatively better than it did to them on listening back to the recording, and consequently they felt intimidated by the recording. This can be understood in terms of a “reality check” for people with an unrealistic estimation of their own skills, or a reaction to a sudden realization that prior skills are no longer intact. Either way, these experience triggered emotions that were difficult to process in the participants’ current life situations. In this respect, music therapists need to exercise a high degree of reflexivity and caution when recording music with this group of patients. We must emphasize, however, that such singular episodes of negative experiences in the context of an overall positive experience call for qualified and mindful music therapy practice, rather than disfavoring music therapy in work with people diagnosed with psychosis.

The most conspicuous finding in this study was that, despite the overall positive user experiences of music therapy as supportive and helpful, several of the participants did not consider music therapy to be a treatment, instead emphasizing its representation of freedom from illness, stigma, and treatment. This was connected to experiences of music being one of the few illness-free spaces or zones in their lives. In consequence, several of our participants expressed strong opposition toward the mixing of music and psychiatric treatment. Thus, it seemed that for many of the participants, music therapy aligned with a precious area in their life that they experienced as outside the realm of illness and treatment. To avoid intruding upon or damaging such a “musical sanctuary”, we need to be open to the idea that for some patients, and perhaps especially those with a particular interest in music, music therapy may not always be appropriate. The findings in this study, however, indicate that music therapy may align with and support the health-promoting use of music by “nurturing” such a freedom zone of engagement with music. Moreover, it seems that for many of our participants it was exactly within this paradox “opposite of treatment” that they found music therapy useful. However, for this to be possible, it is crucial to acknowledge the risks of “pathologizing” or “medicalizing” music if music therapy is framed within a clinical context of assessment or diagnosis-specific interventions (Rolvsjord, 2010).

There is no consensus as to how music therapy processes should be understood in terms of what helps or what the “mechanisms of change” are (Gold et al., 2013). Outcome studies primarily adhere to a pathogenic paradigm, where mental health is understood as the absence of pathology (Provencher & Keyes, 2013). Thus, symptom reduction and functional improvement (Mössler et al., 2011, p. 7) and the relation between the interventions and symptoms (de l’Etoile, 2002) are generally the focus of attention. Our analysis of firsthand accounts demonstrates that participants were generally less concerned with how music therapy reduced symptoms and increased functioning, instead emphasizing more experiences related to their own well-being, hope, or meaning, and indeed sometimes even resisting the very notion of music therapy as concerned with illness and treatment. These factors are all highlighted as important for mental health recovery (Leamy, Bird, Le Boutillier, Williams, & Slade, 2011; Provencher & Keyes, 2013). More traditional “clinical outcomes” (such as decreases in anxiety and disturbing thoughts) were mentioned by some of the participants, but these had the character of being of secondary importance. This harmonizes with Davidson et al.’s (2009) understanding of the relationship between mental health recovery and clinical recovery:

Processes involved in a person’s entering into and being in recovery include the processes that appear to lead to a reduction in the illness as well. The opposite, however, would not necessarily be true. In other words, if we begin with describing the processes involved in minimizing deficit, disability, and dysfunction, we may never arrive at a process of maximizing the person’s health and everyday life. Beginning with the person’s everyday life and his or her efforts to live with the illness, on the other hand, naturally extends to efforts to minimize the illness as disrupting or posing barriers to that life. In brief, minimizing illness is not the same as maximizing health, and our model must incorporate both. (p. 34)

Such a perspective might shed light on the paradoxical “opposite of treatment” health-promoting experiences of our participants. In our study, the participants found themselves “treated” by a professional to a certain extent, but they nevertheless experienced music therapy as an invitation into a non-clinical arena for music making that made them feel more “normal” and well. This points to a discrepancy between how participants in our study experienced music therapy, and the top-down rationale represented in many outcome studies and clinical models, where professionals provide methods, techniques, and interventions aimed at the improvement of the patient’s symptoms and functioning (Odell-Miller, 2007).

Hence, an understanding of music therapeutic processes as a matter of mechanisms changing something within the client may not be the most fruitful approach, partly because it ignores the patient’s own participation and contributions (Rolvsjord, 2010, 2013; Stige, 2012b), and partly because it ignores the potentials of the freedom space so much appreciated by our participants. We argue that the notion of *agency* is a better point of departure for understanding why music therapy is found to be helpful for persons with severe mental illness. Agency can be understood as “the perceived ability to affect one’s own destiny and to engage meaningfully with others and reflects the dimensions of mastery and positive relationships with others” (Provencher & Keyes, 2013, p. 285). Theoretical perspectives in music therapy suggest that the musical interaction in music therapy may offer potentials for such experiences (Abrams, 2012; Ruud, 1997, 2010). Further, these elements of agency correspond to a high degree with our findings in this study, where participants reported that music therapy was an arena for engagement, mastery, and relationship. By *playing* music, *writing* songs, *recording* CDs, and *performing* music, music therapy became an arena for *making* sound, *taking* space, *feeling* alive, and *being* somebody – processes in which the participants regained experiences of ownership and authorship of their thoughts, feelings, and actions (Lysaker & Leonhardt, 2012). According to Davidson and Strauss (1992), entering the role of active agent in one’s own life and becoming a more social human being is interlinked with the process of discovering the possibilities of possessing a more positive and active sense of self. Thus, the recovery perspectives emphasize a radical level of user participation related to service provision (Slade, 2009). Linked to such ideas of user participation, the possibilities to promote clients agency in music therapy have political implications regarding power relations and civil rights (Baines, 2013; Edwards, 2006; Solli, 2012; Stige, 2006).

To enhance agency is empowering and regarded as a cornerstone of hope (Allen, Munich, & Rogan, 2004), which is, in turn, seen as a vital aspect of an ongoing recovery process (Slade, 2009). By maintaining a primary focus on the development of a positive sense of self and identity, and on promoting active and responsible roles in music therapy (and in life), music therapists will be able to support processes of personal and social recovery, something that also leads to symptom relief and better functioning (Davidson et al., 2009). As mental health care worldwide seems to be starting to adapt to the perspective of mental health recovery (Slade et al., 2012), and promotion and protection of mental health as a positive state is becoming a new goal (Fledderus et al., 2010; Herrman et al., 2005), so working within these rationales should make music therapy an attractive approach to help people diagnosed with psychosis in years to come. This could make music therapy relevant in a range of settings, from acute wards, via diverse service centres, to non-clinical community settings.

## Conclusion

We have outlined four main themes of informants’ experiences of their participation in music therapy. Our study confirmed many of the positive findings from outcome studies, but diverged as to how music therapy should be presented to best help patients in their current life situations. The perspective of mental health recovery was found to align to a substantial degree with the participants’ view of what helps and hinders processes of personal and social recovery. We have argued that mental health recovery, with its particular focus on personal and social agency, is a better framework for music therapy for patients with psychosis than a primary focus on symptom remission and elevation of functions. Based on the current findings, we argue for a shift in focus away from illness reduction toward promotion of positive mental health and wellness, away from a primary focus on methods, techniques, and interventions toward music as freedom to be healthy, and away from standardized procedures and manuals toward a primary focus on personal and social agency.

## Funding

This project has been financially supported by the Norwegian ExtraFoundation for Health and Rehabilitation through EXTRA funds, and by Lovisenberg Diakonale Hospital.
